# Dermatoscopic, Histological and Confocal Microscopic Analysis of a Kissing Nevus of the Penis

**DOI:** 10.3390/dermatopathology10020022

**Published:** 2023-05-31

**Authors:** Alexander Bianchi, Alfonso Baldi, Andrea Farabini, Lorenzo Nibid, Vincenzo Roberti, Giovanni Pellacani, Dmitry V. Kazakov, Michele Donati

**Affiliations:** 1 Fondazione Policlinico Tor Vergata, Tor Vergata University, 81-00133 Rome, Italy; 2 Department of Environmental, Biological and Pharmaceutical Sciences and Technologies, University of Campania “L. Vanvitelli”, 43-81100 Caserta, Italy; 3 Istituto di Medicina e Scienza dello Sport “Antonio Venerando”, 1-00197 Rome, Italy; 4 Anatomical Pathology Unit, Department of Medicine and Surgery, Università Campus Bio-Medico di Roma, Via Alvaro del Portillo, 200-00128 Rome, Italy; 5 Dermatology Clinic, Sapienza University of Rome, 155-00161 Rome, Italy; 6 IDP Institut für Dermatohistopathologie, Pathologie Institut Enge, Hardturmstrasse, 133-8005 Zurich, Switzerland; 7 Anatomical Pathology Unit, Fondazione Policlinico Universitario Campus Bio-Medico, Via Alvaro del Portillo, 200-00128 Rome, Italy

**Keywords:** kissing nevus, divided nevus, split nevus, penis, confocal microscopy, dermoscopy, histology

## Abstract

Kissing nevus is a congenital melanocytic neoplasm arising in those parts of the body that split at some point during embryological development (i.e., eyelid; penis), resulting in two adjacent melanocytic nevi. To date, 23 cases of kissing nevus of the penis have been described, and dermatoscopic and histological findings are available in 4/23 cases. We report a dermatoscopic, histological and confocal microscopic analysis in a new case of the kissing nevus of the penis in a 57 years old man. Dermatoscopic analysis showed large globules in the central area and a peripheral pigment network; the histological examination confirmed the presence of an intradermal melanocytic nevus with minimal junctional component and congenital features. Moreover, we reported, for the first time, confocal microscopy findings in the kissing nevus of the penis, revealing the presence of dendritic cells in correspondence with the epidermis and suggesting a state of cellular activity. Considering the clinicopathological features of the lesion, a conservative approach was adopted, and a clinical follow-up was planned after six months.

## 1. Introduction

Kissing (or divided) nevus is a peculiar congenital melanocytic neoplasm arising in those parts of the body that split at some point during embryological development, resulting in two adjacent melanocytic nevi. The first description of this phenomenon was by Von Michael in 1908 in the eyelid, and Fuchs [1] first used the name. Several kinds of kissing nevi have been described involving less common locations than the eyelids. In detail, other reported variants are the divided nevus spilus of the eyelids [2], divided mast cell nevus [3], and a divided form of epidermal nevi of the fingers [4]. Nevertheless, several cases have been reported involving the glans penis and inner foreskin. To date, 23 cases of kissing nevus of the penis have been described [5,6,7,8,9,10,11,12,13,14,15,16,17,18,19,20,21]. Both dermatoscopic and histological data are only available in four cases [16,17,19,21].

The mechanism responsible for the formation of kissing nevus of the eyelids can be explained in relation to the embryologic formation of these structures. The eyelids start to form at weeks 5~6 of gestation and fuse at weeks 8 to 9 of gestation, then split during the 24th week of gestation [22]. Therefore, a kissing nevus of the eyelids may originate between weeks 8 and 24 of gestation when the eyelids are fused; melanoblasts are present at the split border between the upper and lower eyelids. Afterward, with the advancement of cellular division, the two eyelids are separated, and one nevus becomes two lesions located on adjacent sites, resulting in a kissing nevus.

A similar mechanism has been proposed for the kissing nevus of the penis [6,7]. Indeed, around 11~14 weeks of gestation, it is possible to identify two different invaginations in the digital edge of the penis; the epithelial glandular placode that generates the glandular urethra and the epithelial preputial placode that splits and gives origin to the glans and the prepuce. Two slightly different mechanisms have been hypothesized for the formation of the kissing nevus of the penis. Desruelles et al. hypothesized how melanoblasts and melanocytes migrate to the prepuce around the 12th week and form the melanocytic lesion before its separation from the glans. After the separation, each nevus may grow autonomously [7]. In contrast, Kono et al. proposed that melanoblasts begin to migrate just after the conclusion of the invagination of the preputial epithelial placode [6]. This theory could also explain the preferred location of the lesions in the dorsal or dorsolateral aspect of the penis since the epithelial invagination from the dorsal side precedes the ventral side.

We herewith report a new case of the kissing nevus of the penis, describing for the first time the dermatoscopic and histological features and confocal microscopic findings.

## 2. Case Report

**Figure 1:**
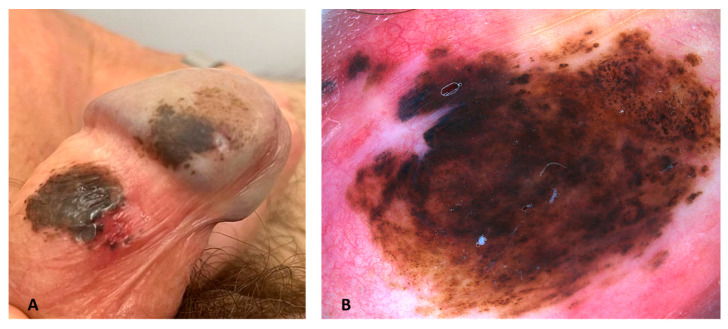
(**A**) Clinical and (**B**) Dermoscopic presentation of “kissing nevus”. The lesion presented large globules in the central area and a peripheral pigment network. Heine Delta 30 dermoscopy (HEINE Optotechnik GmbH & Co., Gilching, Germany).

A 57-year-old man presented for a dermatological examination of two adjacent pigmented lesions on the glands and the inner foreskin that had been present for a long time, growing slowly during the years (Figure 1A). The two lesions were well-defined oval-shaped pigmented macules with a color ranging from brown to black and with a smooth surface. They presented as two mirrored nevi, symmetrical in relation to the coronal sulcus. The dermoscopic analysis of the lesions displayed a pattern characteristic of a compound melanocytic nevus with large globules in the central area and a peripheral pigment network (Figure 1B).

**Figure 2:**
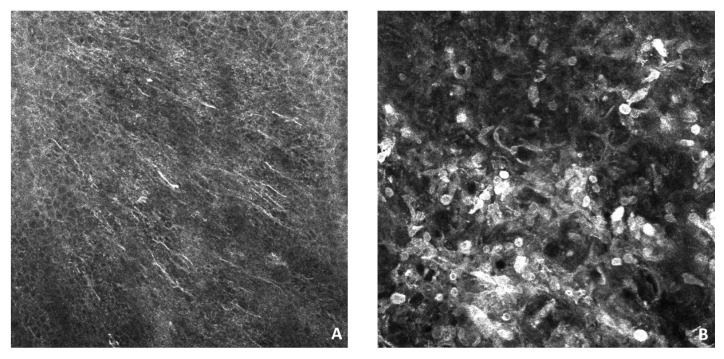
Reflectance confocal microscopy examination shows (**A**) The presence of intraepithelial bright, hyper-reflecting dendritic cells and (**B**) Nests of aggregated melanocytes with some large, pleomorphic cells in papillary dermis. VIVASCOPE 1500 (VivaScope GmbH, Munich, Germany).

Confocal microscopy demonstrated the presence of various dendritic cells in correspondence with the epidermis, which is indicative of the state of cellular activity (Figure 2).

Two incisional biopsies were performed in both involved anatomical sites in order to exclude malignancy and to consider a conservative treatment. The biopsies were fixed in formalin and embedded in paraffin, following standard protocols. Paraffin sections were cut at 5 µm using a microtome LEICA SM 2000 R (Advanced Research Systems Inc., Macungie, PA, USA), dewaxed in xylene, rehydrated through a series of graded ethanol solutions and stained with Gill’s Hematoxylin and Eosin (Bio-Optica, Via San Faustino 58-20134 Milan, Italy). Immunohistochemistry was executed on an automated immunostainer (Bond-III, Leica, Biosystems, Buccianasco, Italy), as previously described [23,24]. The primary antibodies used were Melanoma Marker HMB45 (clone HMB45) and Microphthalmia Transcription Factor (clone 34CA5) (Leica, Biosystems, Buccianasco, Italy). Images were obtained using the NanoZoomer S360 digital slide scanner (Hamamatsu Photonics, Hamamatsu, Japan).

**Figure 3:**
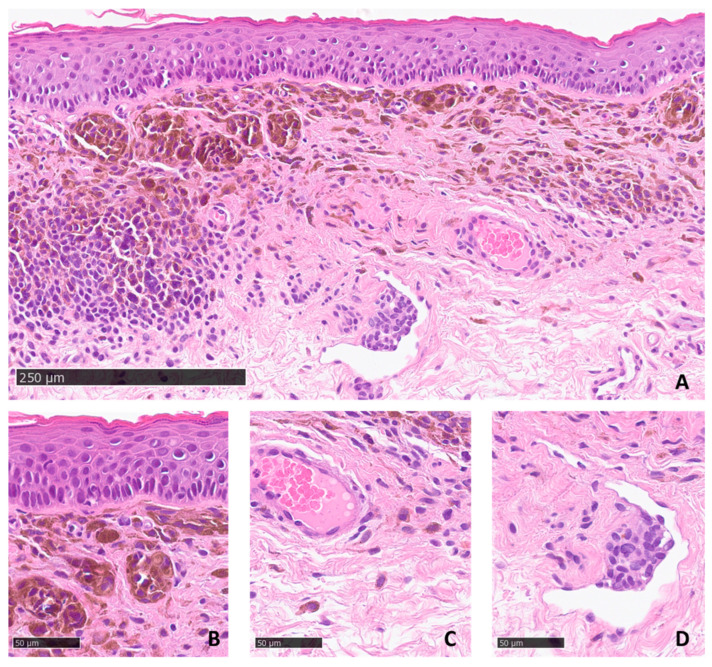
(**A**) Histological examination revealed a melanocytic nevus with congenital features associated with abundant stromal melanin deposits, scale bar: 250 µm. (**B**) Close-up view showing melanocytic nests in the upper dermis, (**C**) Single melanocytes interspersed in the reticular dermis. (**D**) Note that melanocytic aggregates protruded slightly in a dilated lymphatic vascular channel, scale bar: 50 µm. NanoZoomer S360 digital slide scanner (Hamamatsu Photonics, Hamamatsu, Japan).

The histological examination of the samples revealed similar microscopic features in both samples, showing an intradermal melanocytic proliferation with congenital features and a minimal junctional component. Melanocytes were arranged in nests in the upper dermis while splaying between dermal collagen bundles in the reticular dermis. Stromal melanin deposits were more abundant in the upper part of the lesion, as were dermal melanophages. Melanocytic aggregates protruding within a vascular channel with an empty and dilated lumen lined by flattened endothelial cells were also observed (Figure 3). Immunohistochemical analysis using antibodies for Melanoma Marker HMB45 and Microphthalmia Transcription Factor confirmed that the neoformation was of melanocytic origin (data not shown). Based on the clinical and histological findings, the diagnosis of kissing nevus of the penis was rendered, and a conservative approach was adopted. A clinical follow-up was planned at six months.

## 3. Discussion

The kissing (or divided) nevus of the penis is a rare entity, with only 23 cases reported. The age at diagnosis varies from 3 to 30 years old [18,19]. These lesions can be congenital and noticed at birth or have a late onset and be initially noticed during puberty. To the best of our knowledge, both dermoscopic and histological data were available in only 4/23 reported cases of divided nevus of the penis (Table 1) [16,17,19,21].

The dermoscopic examination of this variant of melanocytic nevus generally revealed a globular or composite pattern (globular-reticular pattern or pigment network at the periphery and homogeneous pattern with some globules in the center) [16,17,19,20,21].

Microscopically, kissing nevi are compound or intradermal melanocytic neoplasms that are generally associated with numerous intradermal melanophages [5,6,8,9,10,11,12,14,15,16,17,19,21]. To date, only one case of melanoma arising in congenital kissing nevus has been reported; in this unique case, the term kissing melanoma has been proposed [18].

We described a new case of the kissing nevus of the penis in a 57-year-old man. Dermoscopic analysis, in our case, displayed the characteristic pattern of a compound melanocytic nevus with large globules in the central area and a peripheral pigment network. This pattern was consistent with the previously reported dermoscopic description of kissing nevus of the penis [16,17,19,20,21].

The diagnosis of compound melanocytic nevus was confirmed by histological examination. The two incisional biopsies performed on both involved anatomical sites revealed an intradermal melanocytic proliferation with congenital features and a minimal junctional component. In the upper part of the lesion, we observed abundant stromal melanin deposits together with scattered dermal melanophages. Similar findings were observed in previously reported cases of the kissing nevus of the penis [14,15]. Interestingly, we also noticed melanocytic aggregates protruding into an empty and dilated vascular channel lined by flattened endothelial cells, which strongly suggested a lymphatic vessel. Intralymphatic melanocytic aggregates are an uncommon feature of the benign compound and intradermal nevi. These aspects have been defined as “intralymphatic nevus cell protrusion” (ILNP) when melanocytes present as subendothelial hillocks and “intralymphatic nevus cell aggregates” (ILNA) when the endothelial layer lining the periphery of the melanocytic aggregates is observed confirming their intraluminal location [25,26,27,28,29]. It is important to underline that ILNP and ILNA are not a sign of malignancy in melanocytic neoplasms and should not be confused with the lymphovascular invasion observed in malignant melanoma. ILNP and ILNA should also be distinguished from artifactual clefts resulting from tissue processing that mimic lymphatic or vascular spaces. ILNP and ILNA have been observed in Spitz nevi and have more often shown evidence of vascular invasion [30]. To the best of our knowledge, ILNP and ILNA have never been described in the previously reported cases of a kissing nevus. The observation of intralymphatic melanocytic aggregates has no clinical implication and supports the hypothesis that benign nodal melanocytic aggregate likely results from melanocytic emboli that are transferred via lymphatics to the draining lymph node [27].

Moreover, we reported, for the first time, confocal microscopy findings in a kissing nevus of the penis; the analysis revealed the presence of several intra-epidermal dendritic cells, suggesting a state of cellular activity. An increased number of epidermal dendritic cells was proposed as an independent risk factor for melanoma and can represent a diagnostic pitfall in the kissing nevus of the penis, although further data are needed to confirm this finding [31].

The great majority of the kissing nevi of the penis described are benign lesions. For cosmetic reasons, surgical excision and reconstruction by skin grafting using remnant foreskin have been performed with satisfactory results [10]. To date, only one case of the malignant transformation of the kissing nevus of the penis has been reported [18]. Malignant melanoma of the penis is extremely rare, representing less than 2% of primary penile malignancies [30]. In our case, the dermoscopic and histological features of the kissing nevus suggested a benign melanocytic lesion. Therefore, to avoid the possibility of a scar and deformity of the glans penis following a surgical procedure, we chose a prudential approach with a follow-up control at six months.

## Figures and Tables

**Table 1:** Cases of kissing nevi of the penis described in the English literature in which both dermatoscopic and histological data are available.

Author	Age at the Diagnosis (y)	Dermoscopy	Histology
Mendes et al. [16]	11	Globular pattern: multiple pigment globules of different size	Compound melanocytic nevi
Alves de Souza et al. [17]	9	Compound pattern: fine pigmented network (periphery) and darkened globules of multiple size (center)	Compound melanocytic nevi
Savas et al. [19]	3	Globular pattern: scattered pigmented globules of varying size on a pigmented area with a pigmented center and dark periphery	Compound melanocytic nevi
Correia et al. [21]	14	Compound pattern: pigmented patch with dark dots and discrete annular-granular structures (prepuce) and a darker pigmented patch with greater density dark dots (glans)	Compound melanocytic nevi
Our case	57	Compound pattern: fine pigmented network (periphery) and darkened globules of multiple size (center)	Compound melanocytic nevi

## Data Availability

Not applicable.

## References

[1] Fuchs A. (1919). Ueber geteilte naevi der augenlider. Klin. Mon. Augenheikd.

[2] Sato S., Kato H., Hidano A. (1979). Divided nevus spilus and divided form of spotted grouped pigmented nevus. J. Cutan. Pathol..

[3] Niizawa M., Masahashi T., Maie O., Takahashi S. (1989). A case of solitary mastocytoma suggesting a divided form of mast cell nevus. J. Dermatol..

[4] Hayashi N., Soma Y. (1993). A case of epidermal nevi showing a divided form on the fingers. J. Am. Acad. Dermatol..

[5] Phan P.T., Francis N., Madden N., Bunker C.B. (2004). Kissing naevus of the penis. Clin. Exp. Dermatol..

[6] Kono T., Nozaki M., Kikuchi Y., Erçöçe A.R., Hayashi N., Chan H.H.L., Ohkubo R., Fukaya E. (2003). Divided naevus of the penis: A hypothesis on the embryological mechanism of its development. Acta Derm. Venereol..

[7] Desruelles F., Lacour J.P., Mantoux F., Ortonne J.P. (1998). Divided nevus of the penis: An unusual location. Arch. Dermatol..

[8] Yun S.J., Wi H.S., Lee J.B., Kim S.J., Won Y.H., Lee S.C. (2011). Kissing Nevus of the Penis. Ann. Dermatol..

[9] Zhou C., Xu H., Zang D., Du J., Zhang J. (2010). Divided nevus of the penis. Eur. J. Dermatol..

[10] Higashida Y., Nagano T., Oka M., Nishigori C. (2010). Divided naevus of the penis. Acta Derm. Venereol..

[11] Palmer B., Hemphill M., Wootton C., Foshee J.B., Frimberger D. (2010). Kissing nevus discovered at circumcision consult. J. Pediatr. Urol..

[12] Wang S., Zhou M., Qiao J. (2014). Kissing nevus of the penis. Report of two cases and review of the literature. An. Bras. Dermatol..

[13] Li Z.B., Liu T., Zhang Q.G., Hu J.T. (2015). Treatment of Divided Nevus of the Penis with Circumcision and Free Inner Prepuce Grafting. Plast. Reconstr. Surg. Glob. Open..

[14] Hardin C.A., Tieu K.D. (2013). Divided or kissing nevus of the penis. Derm. Online J..

[15] Choi G.S., Won D.H., Lee S.J., Lee J.H., Kim Y.G. (2000). Divided naevus on the penis. Br. J. Dermatol..

[16] Mendes C.P., Samorano L.P., Alessi S.S., Nico M.M.S. (2014). Divided naevus of the penis: Two paediatric cases with dermoscopic findings. Clin. Exp. Dermatol..

[17] Alves de Souza N.G., Nai G.A., Schaefer A.L.F., Schaefer L.V. (2017). Kissing nevus of the penis: A case report and dermatoscopic findings. An. Bras. Dermatol..

[18] Egberts F., Egberts J.H., Schwarz T., Hauschild A. (2007). Kissing melanoma or kissing nevus of the penis?. Urology.

[19] Savas S., Balı Y.Y., Erdemir A.V., Simsek H. (2018). Divided nevus of the penis. Int. J. Dermatol..

[20] Armengot-Carbó M., Rodrigo-Nicolás B., Botella-Estrada R. (2018). Divided or kissing nevus of the penis: A new case with dermoscopic findings. Pediatr. Dermatol..

[21] Correia B., Duarte A.F., Haneke E., Correia O. (2021). CO2 laser treatment of a kissing nevus of the penis: An alternative solution for a rare condition. J. Dermatolog Treat..

[22] Guerra-Tapia A., Isarría M.J. (2005). Periocular vitiligo with onset around a congenital divided nevus of the eyelid. Pediatr. Dermatol..

[23] Spugnini E.P., Menicagli F., Giaconella R., Zanni F., Camponi C., De Luca A., Santoro A., Baldi A. (2021). Filling the gap between histology and cytology: Description of an innovative technology (Cytomatrix) to increase the diagnostic effectiveness of fine needle aspirates data. J. Clin. Pathol..

[24] Bonucci M., Minelli S., Castro C.L., Camponi C., Scimeca M., Scipioni A., Spugnini E.P., Baldi A. (2021). Cytomatrix, a new procedure to enhance the diagnostic usefulness of fine needle aspirates. Ann. Res. Oncol..

[25] Sood N., Mukherjee M. (2018). Dermal Lymphatic Invasion: A Rare Feature in Benign Intradermal Nevus. Int. J. Appl. Basic. Med. Res..

[26] Leblebici C., Kelten C., Gurel M.S., Hacıhasasanoglu E. (2016). Intralymphatic nevus cells in benign nevi. Ann. Diagn. Pathol..

[27] Bell M.E., Hill D.P., Bhargava M.K. (1979). Lymphatic invasion in pigmented nevi. Am. J. Clin. Pathol..

[28] Katsumata M., Matsunaga T., Maruyama R., Ezoe K. (1990). Lymphatic invasion of nevus cells observed in intradermal nevus. J. Dermatol..

[29] Howat A.J., Variend S. (1985). Lymphatic invasion in Spitz nevi. Am. J. Surg. Pathol..

[30] Demitsu T., Nagato H., Nishimaki K., Okada O., Kubota T., Yoneda K., Manabe M. (2000). Melanoma in situ of the penis. J. Am. Acad. Dermatol..

[31] Guiducci L., Kaleci S., Chester J., Longo C., Ciardo S., Farnetani F., Pellacani G. (2022). Dendritic cells in reflectance confocal microscopy are a clue for early melanoma diagnosis in extrafacial flat pigmented melanocytic lesions. Exp. Dermatol..

